# A motivational framework of acculturation

**DOI:** 10.1002/brb3.2267

**Published:** 2021-06-24

**Authors:** Allon Vishkin, Gabriel Horenczyk, Pazit Ben‐Nun Bloom

**Affiliations:** ^1^ Institute for Social Research University of Michigan 500 S. State Street Ann Arbor Michigan 48109 USA; ^2^ Department of Education The Hebrew University of Jerusalem Mt. Scopus Jerusalem 91905 Israel; ^3^ Department of Political Science The Hebrew University of Jerusalem Mt. Scopus Jerusalem 91905 Israel

**Keywords:** acculturation, goal pursuit, migration, motivation, well‐being

## Abstract

A key distinction in motivational processes is between motivations and the means for pursuing motivations. Despite being a motivated process, existing models of acculturation do not make this distinction, neither empirically nor theoretically. A motivational framework that is informed by theories of goal constructs to understand the process of acculturation is proposed. This model is tested in two distinct samples comprising immigrants from the former Soviet Union to Israel (*N* = 239) as well as immigrants from Pakistan, India, and Bangladesh to Britain (*N* = 236). Results revealed that the motivation to preserve one's heritage culture and the motivation to adopt one's host culture were each uniquely associated with the respective means for doing so. Furthermore, outcomes in acculturation were determined by the match between acculturation motivations and acculturation means. These findings demonstrate the theoretical and practical implications of analyzing the process of acculturation using a motivational framework.

## Abstract 2

Despite being a motivated process, existing models of acculturation do not distinguish between motivations in acculturation and means in acculturation. We test a motivational framework that is informed by theories of goal constructs to understand the process of acculturation.

## 1 INTRODUCTION

Worldwide rates of immigration have risen significantly in the past decade (United Nations, Department of Economic and Social Affairs/Population Division, 2015). The integration of large immigrant populations requires a nuanced understanding of immigrants’ motivations. Immigrants may be motivated to assimilate to the host culture, to preserve their heritage culture, or both. All immigrants are likely to pursue means that facilitate the attainment of their respective motivations, such as adopting (or preserving) their host (or heritage) language, and acquiring social contacts from the communities of their host (or heritage) culture. Existing models of acculturation account for these two types of pursuits under the label of acculturation strategies (Berry, 1997, 2003). Yet, such models do not fully distinguish, empirically or theoretically, between motivations in acculturation and means in acculturation. Such a distinction is critical to understanding the process of acculturation, and ultimately to shaping policies and interventions that seek to incorporate immigrants successfully in their new countries. For instance, if outcomes in acculturation are dependent on the presence of both acculturation motivations and the means for enacting these motivations, then successful policies and interventions must take into account both of these elements. To address this limitation, we propose a motivational framework of acculturation that distinguishes between motivations and means in acculturation, informed by theories of goal constructs (Austin & Vancouver, 1996; Kruglanski et al., 2002; Kruglanski et al., 2015). We test this framework in two distinct immigrant samples.

### 1.1 Goal constructs: Distinguishing between motivations and means

Theories of goal constructs address how people translate goals into concrete actions for the purpose of attaining them (Austin & Vancouver, 1996; Kruglanski et al., 2002; Kruglanski et al., 2015). These theories distinguish between the goals that people pursue and the means for pursuing those goals. Relations between specific goals and specific means are such that a set of means is instrumental to attaining each goal, and a set of goals is attained by each means. For example, a college student's goal of achieving high grades is facilitated by the means of studying for a test or getting a good night's sleep, whereas her goal of socializing is facilitated by the means of going to a party. Thus, the set of means for pursuing a goal of achieving high grades (studying, getting a good night's sleep) differs for the set of means for pursuing a goal of socializing (partying).

Another implication of a goal constructs framework is that outcomes are determined by engaging in means that promote one's goals. For instance, a student who attempts to achieve high grades by going to a party the night before an exam is less likely to reach her goal than a students who attempts to achieve the same goal by studying the night before an exam. We leverage these insights regarding the properties of goal constructs to understand the role of motivations and means in acculturation.

### 1.2 Motivations and means in acculturation

Individuals who move from one culture to another engage in a process called acculturation, in which immigrants experience psychosocial changes as a result of the transition between socio‐national contexts (Berry, 2003; Castro, 2003; Sam & Berry, 2010). Early models of acculturation presented it as a unidimensional process by which immigrants blend into the host culture in terms of values, social contacts, identification, attitudes, behavior and civic assimilation, while foregoing one's original culture (Gordon, 1964). Later models presented acculturation as bi‐dimensional, pertaining to the retention or rejection of (1) one's native culture and (2) one's host culture. Crossing these two aspects results in four hypothesized acculturation strategies: adopting the host culture and rejecting one's original culture (assimilation), rejecting the host culture and maintaining the culture of origin (separation), accepting both (integration) or rejecting both (marginalization; Berry, 2003). Other models divide marginalization into anomie and individualism (Bourhis et al., 1997). Within these frameworks, integration is viewed as the most adaptive acculturation strategy, and marginalization as the least so.

The process of acculturation necessarily sparks two value judgments (Bourhis et al., 1997; LaFromboise et al., 1993; Ryder et al., 2000) that translate into acculturation motivations: the extent to which one wants to preserve or reject one's heritage culture, and the extent to which one wants to adopt or reject their host culture. According to theories of goal constructs, these disparate motivations have downstream consequences for behavior because the means for attaining different motivations vary (Austin & Vancouver, 1996; Kruglanski et al., 2002). For example, intentional exposure to one's heritage language, such as by reading literature or watching movies in one's heritage language, may serve as a means for preserving one's heritage culture, whereas exposure to the host language may serve as a means to adopting one's host culture. Indeed, among Spanish immigrants to Canada, those who were motivated to adopt the host culture displayed better English language proficiency (Masgoret & Gardner, 1999). In addition, maintaining social contacts with members of one's heritage culture may serve as a means to preserving one's heritage culture, whereas possessing social contacts with members of one's host culture may serve as a means to adopting one's host culture. Indeed, among Korean immigrants to the United States, the amount of social contact with members of the host culture predicted greater adoption of the host culture, as assessed by emotional acculturation (De Leersnyder et al., 2011).

**FIGURE 1:**
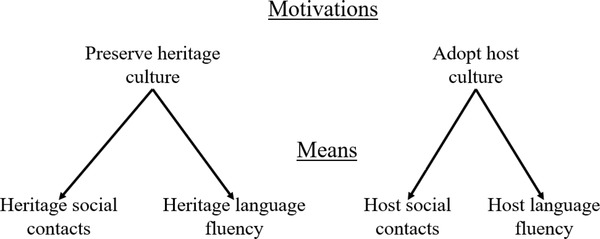
Relations between motivations in acculturation and means in acculturation

This analysis points to a simple set of relations between motivations in acculturation and means in acculturation with important theoretical implications. As depicted in Figure 1, different acculturation motivations are associated with a different set of means for attaining them. Before outlining a set of predictions based on these relations, we first address how motivations and means in acculturation relate to existing constructs in acculturation.

### 1.3 Relations to existing constructs in acculturation

The distinction between motivations in acculturation and means in acculturation has not been examined either theoretically or empirically. These constructs bear some resemblance to other constructs in acculturation, yet are distinct from them. Previous research has recognized the role of motivation in processes related to acculturation. Much research has examined what motivates immigrants to leave one community or country in favor of another (Carling & Collins, 2018; Harris & Todaro, 1970; Lee, 1966). Additional research has examined how different motivations of sojourners when travelling abroad affect their adaptation in the host culture (Chirkov et al., 2008; Chirkov et al., 2007.) These motivations, sometimes called push factors and pull factors, have been identified as factors that occur prior to acculturation (Berry, 1997). However, the applicability of this research to acculturation is limited. In particular, push and pull factors focus on motivations that are in place before arrival in the host country. A potential immigrant who possesses the motivation to leave her home country or to arrive in another country satisfies that motivation upon arriving in that country. Following arrival in a new country, motivations in acculturation come into play. For instance, a group of immigrants may arrive in a new country due to the same set of push and pull factors, but individuals in that group may still vary in their motivations to adopt the host culture or to preserve their heritage culture. Thus, motivations in migration are distinct from motivations in acculturation and occur at different phases in the lives of immigrants.

Previous empirical and theoretical work in acculturation has meshed together motivations and means in acculturation. Empirically, measures of acculturation assessed both motivations and means in a single measure. For example, the Vancouver Index of Acculturation (Ryder, et al., 2000) includes in a single measure motivations in acculturation (e.g. It is important for me to maintain or develop the practices of my heritage culture) as well as means in acculturation (e.g. I often participate in my heritage cultural traditions). Theoretically, Berry (1997, 2003) has distinguished four types of acculturation strategies based on whether people accept or reject their heritage or host culture. However, acculturation strategies as identified by Berry (2003) consist of both the preferences for acculturation and the behavioral outcomes of these preferences, without a clear distinction between the two. Thus, in the existing literature on acculturation, the distinction between motivations and means is still waiting for theoretical and empirical elaboration.

### 1.4 Predictions from a motivational framework of acculturation

Identifying a set of relations between motivations and means in acculturation is useful for advancing several hypotheses. First, immigrants are likely to pursue means that help them attain their motivations (see Figure 1). This means that immigrants are likely to engage in behaviors that promote the attainment of their acculturation motivations, and not engage in behaviors that do not promote their acculturation motivations. Second, because successful goal pursuit requires means for attaining one's goals, outcomes in acculturation are likely to be determined by the match between immigrants’ acculturation motivations and acculturation means. For example, the motivation to adopt the host culture is likely to contribute to adaptive outcomes only to the extent that the means for doing so are available. Thus, two immigrants with the identical motivation to adopt the host culture may differ in how successfully they acculturate, such that the immigrant with more means for adopting the host culture will be more successful in doing so than the immigrant with less means for adopting the host culture. This observation is capable of resolving inconsistencies in the acculturation literature. For example, the extant literature has found inconsistent associations regarding the link between social contacts and positive outcomes in acculturation. Some studies have found that maintaining social contacts with members of the heritage culture is related to higher well‐being (e.g. Vega et al., 1991; Ward & Kennedy, 1993). On the other hand, other studies have found that possessing social contacts with members of the host culture is related to higher well‐being (Berry et al., 1987; Kealey, 1989). To the extent that well‐being is associated with attaining personally valued goals (e.g. Oishi et al., 1999), possessing social contacts from the heritage or host culture should contribute to well‐being when those contacts help reach one's motivations in acculturation. Consequently, maintaining social contacts with members of the heritage culture will likely contribute the most to well‐being when one is motivated to preserve one's heritage culture, whereas possessing social contacts with members of the host culture will likely contribute the most to well‐being when one is motivated to adopt the host culture.

In the present investigation, we examined these two predictions in two samples of immigrants. In particular, we first tested whether people pursue means in acculturation that are congruent with their motivations in acculturation. Next, we tested whether motivations and means that are congruent interact to predict outcomes in acculturation. We focused on outcomes related to well‐being, including life satisfaction and depression, which have been studied frequently as outcomes in acculturation research (e.g. Berry & Kim, 1988; Berry et al., 2006; Jang & Chiriboga, 2011; Noh & Kaspar, 2003; Oei & Notowidjojo, 1990; Ryder et al., 2000; Stuart et al., 2020). To be able to generalize the findings, we selected samples of immigrants with disparate immigration profiles.

Study 1 consisted of immigrants from the former Soviet Union to Israel who immigrated by virtue of the Israeli Law of Return and thus represent a type of diaspora migration (Silbereisen et al., 2014). These immigrants either come from a Jewish background, though many may not be formally recognized as Jewish by Orthodox Jewish law, or have family members with a Jewish background, such as by marriage, but are not Jewish themselves. By moving to Israel, most of these immigrants become a part of the majority religion. In general, these immigrants have sought to maintain a bicultural identity in which they preserve their heritage culture, while simultaneously adopting the host culture (Horenczyk & Bergman, 2016). The host culture, Israel, has an inviting attitude towards Jewish immigrants (Bourhis & Dayan, 2004).

Study 2 consisted of Indian, Pakistani, and Bangladeshi immigrants to the United Kingdom. In contrast to immigrants from the former Soviet Union to Israel, these immigrants are not a type of diaspora immigration. Most of these immigrants come from a Muslim or Hindu background, and by moving to Britain, they become a religious minority. These immigrants endorse, in general, more positive attitudes towards preserving their heritage culture than adopting the host culture (Brown et al., 2016). The host culture, Britain, has a more ambivalent attitude towards immigrants in terms of policy and prevailing attitudes. Thus, the samples in Studies 1 and 2 differ in terms of the heritage culture, host culture, type of immigration, and immigration attitudes of the host culture. The differences between samples enable us to test how well a motivational framework of acculturation generalizes across immigrant groups. Research was conducted in line with the APA Code of Conduct and received ethics approval from the Institution's Internal Review Board. Data and syntax for both studies are available via the Open Science Framework (https://osf.io/4z7m9).

## 2 STUDY 1

In Study 1, we tested a motivational framework of acculturation among immigrants from the former Soviet Union to Israel. The survey was administered in Hebrew to immigrants who immigrated to Israel at least eight years prior.

### 2.1 Method

#### 2.1.1 Participants

The sample comprised participants who immigrated to Israel from regions that were part of the former Soviet Union. An early wave of immigrants from the Soviet Union arrived in the later 1970's, while a larger wave arrived in the years immediately following the breakup of the Soviet Union (Tolts, 2003). This wave consisted of over a million immigrants and constituted approximately 15% of the Israeli population at the time (Horenczyk & Bergman, 2016).

Participants were selected based on identifying as speakers of Russian on an Israeli panel. Since language acquisition of non‐native speakers is similar to the attainment of native bilinguals until around the age of 12 (Hartshorne et al., 2018), we selected participants who were aged 12 or older when they immigrated. Furthermore, since the acculturation of immigrants varies by age of immigration (e.g. De Leersnyder et al., 2011), we sought to recruit a sample as similar as possible in terms of age. In the survey panel used for the present study, it was possible to recruit a sample of sufficient size with a cut‐off age for immigration at 45 years old. In addition, since acculturation experiences vary by the amount of time since immigration, we selected participants who immigrated at least eight years previously.

To achieve sufficient power, we strove to recruit a sample of 200 participants. Such a sample is sufficient to detect a small‐to‐medium effect size of *r* = .20 at 80% power. A total of 242 participants completed the survey. Following norms for monitoring data quality based on completion times, we removed participants who completed the survey in less than one‐third of the median time (e.g. Georgeac et al., 2019; Vishkin et al., 2019), leaving 239 participants (73% female, M_age_ = 37.88, SD_age_ = 6.12).

### 2.2 Materials

#### 2.2.1 Means for acculturation

Participants reported the extent to which they possess two means for acculturation: language skills and social contacts. To assess language skills pertaining to their heritage culture, participants completed four items regarding the extent to which they are capable of understanding, speaking, reading, and writing a letter in Russian (*α* = .86). To assess language skills pertaining to their host culture, participants completed the same items with reference to Hebrew (*α* = .96). Items were assessed on 5‐point scale from 1 (not at all) to 5 (very well). The items in these scales were adapted from Masgoret and Gardner (1999).

To assess social contacts, participants reported the extent to which they have three types of social contacts. First, they reported the extent to which they have friends from Russian or Israeli non‐Russian backgrounds on a 5‐point scale from 1 (none) to 5 (many). Next, they reported how often they spend time with Russians or with non‐Russian Israelis on a 5‐point scale from 1 (almost never) to 5 (always). Finally, they reported the extent to which their neighbors are Russian or non‐Russian Israelis on a 5‐point scale from 1 (none) to 5 (most). The items in these scales were adapted from De Leersnyder et al. (2011). The reliabilities were lower than for the previous scales (Russian social contacts: *α* = .56; Israeli social contacts: *α* = .66), reflecting in part the fewer items in these scales. Removing one item from the measure of Russian social contacts to improve reliability altered only one of the results which we indicate below.

#### 2.2.2 Motivations in acculturation

While means in acculturation assessed current states, motivations in acculturation assessed valued states. To assess acculturation motivations, participants reported the extent to which it is important for them to engage in various cultural practices with regards to their heritage and host culture. For the motivation to preserve heritage culture, these included observing Russian practices and holidays, speaking Russian perfectly, following and staying up‐to‐date with Russian music, preserving and developing their Russian identity, and staying up‐to‐date with news on Russia (*α* = .83). The motivation to maintain their host culture was assessed using the same items, except with reference to Hebrew or Israel (*α* = .80). Items were assessed on 5‐point scale from 1 (not at all) to 5 (to a great extent). The wording for these subscales was adapted from items 13 and 14 of the Vancouver Index of Acculturation (Ryder et al., 2000) that tap motivations in acculturation.

#### 2.2.3 Outcomes

Outcomes in acculturation were examined via two scales assessing well‐being. The Satisfaction with Life Scale (SWLS; Diener et al., 1985) assess life satisfaction and includes 5 items (*α* = .87) rated on a 7‐point scale from 1 (strongly disagree) to 7 (strongly agree). The Center of Epidemiological Studies Depression Scale (CES‐D; Andresen et al., 1994) assesses depressive symptoms and includes 10 items (*α* = .79) rated on a 4‐point scale from 1 (rarely or none of the time) to 4 (most or all of the time). These scales have been used previously to assess outcomes in acculturation (e.g. Berry et al., 2006; Jang & Chiriboga, 2011).

### 2.3 Procedure

The sample was recruited through an Israeli online survey company (www.ipanel.co.il). After giving consent, participants indicated their age at the time of immigration. Only those who immigrated between the ages of 12–45 could complete the rest of the survey. Next, participants completed the measures in the following order: acculturation motivations, language proficiency, social contacts, outcomes, and demographics. Both heritage and host culture motivations, as well as heritage and host culture language proficiency, were presented in a counter‐balanced order. Additional measures not directly related to the present investigation were included, including measures of national identity, political efficacy and trust, physical descriptions of one's neighborhood, and desired emotions.

### 2.4 Results

Table 1 presents the means and standard deviations of the main variables and the zero‐order correlations among these variables. All the heterotrait‐monotrait (HTMT) ratio of correlation values between the two motivations and four means were below .85 (Henseler et al., 2015), demonstrating discriminant validity between the study variables.

**Table 1:** Descriptive statistics and pairwise correlations among study variables (Study 1)

Variable	*M*	*SD*	1	2	3	4	5	6	7
1. Host motivation	3.94	0.69							
2. Host language fluency	4.55	0.61	.34**						
3. Host social network	3.78	0.79	.45**	.31**					
4. Heritage motivation	3.09	0.87	.05	.01	−.09				
5. Heritage fluency	4.85	0.36	.11	.19**	.02	.11			
6. Heritage social network	3.83	0.64	−.05	−.06	−.22**	.44**	0		
7. Life satisfaction	4.57	1.20	.19**	.12	.21**	.06	.01	.10	
8. Depressive symptoms	1.90	0.46	−.13*	−.25**	−.08	.03	−.09	.04	−.36**

Note:

* *p* < .05;

** *p* < .01.

#### 2.4.1 Associations between motivations and means

Zero‐order correlations revealed that the motivation to adopt the host culture is associated with both means for doing so (host language fluency and host social networks), but is unassociated with the means for preserving the heritage culture. Furthermore, the motivation to preserve the heritage culture is associated with the social means for doing so, but not with the linguistic means for doing so or with the means for adopting the host culture. The lack of a significant association between the motivation to preserve the heritage culture and the linguistic means for doing so is most likely due to the ceiling effect for the measure assessing heritage language abilities (*M* = 4.85 on a scale of 1–5).

**FIGURE 2:**
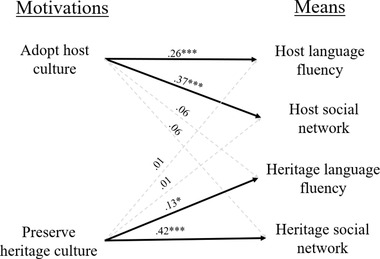
Associations between acculturation motivations and means (Study 1). Note: Values reflect standardized regression coefficients after controlling for demographic variables; *p < .05; **p < .01; ***p < .001

To control for covariates, we regressed each motivation on the four means as well as on several demographic variables, including gender, age, religiosity, and education (see Table 2).^1^ Results revealed that each motivation is associated with, and only with, its respective means (see Figure 2). Moreover, after controlling for demographic covariates, the association between preserving the heritage culture and heritage language fluency became significant.^2^ This was not due to the inclusion of a particular covariate.

**Table 2:** Associations between acculturation motivations and means (Study 1)

	Adopt host culture				Preserve heritage culture			
	*β*	SE	*β*	SE	*β*	SE	*β*	SE
Host language fluency	.21***	0.06	.26***	0.07	.02	0.06	.01	0.07
Host social network	.39***	0.06	.37***	0.06	−.01	0.06	.01	0.06
Heritage language fluency	.06	0.06	.06	0.06	.11	0.06	.13*	0.07
Heritage social network	.05	0.06	.06	0.06	.44***	0.06	.42***	0.06
Gender (M = 1, F = 2)	—	—	.36**	0.13	—	—	−.14	0.13
Age	—	—	.07	0.06	—	—	.06	0.06
Religiosity	—	—	.07	0.06	—	—	−.06	0.06
Education	—	—	−.05	0.06	—	—	−.08	0.06

Note:

* *p* < .05;

** *p* < .01;

*** *p* < .001.

#### 2.4.2 Acculturation outcomes

To test whether motivations and means in acculturation interact to predict outcomes in acculturation, we regressed the two measures of well‐being on acculturation motivations, acculturation means, and their interactions. Since each motivation was associated with both of its means, we collapsed across each type of means by averaging both means associated with the motivation to adopt the host culture and both means associated with the motivation to preserve the heritage culture.^3^ We expected that positive acculturation outcomes would be predicted by the interaction of a motivation with its congruent means (e.g. motivation to adopt the host culture with the means for adopting the host culture), but not by the interaction of a motivation with its incongruent mean (e.g. motivation to adopt the host culture with the means for preserving the heritage culture).

Results revealed, first, that none of the interactions between a motivation and an incongruent mean predicted either life satisfaction or depressive symptoms (Table 3). In addition, the interaction between the motivation to adopt the host culture and the means for doing so predicted both life satisfaction and depressive symptoms. However, both interactions were opposite the expected direction. In particular, greater motivation to adopt the host culture was least conducive to the well‐being of those with the means to do so.

**Table 3:** Predicting outcomes in acculturation via acculturation motivations and means (Study 1)

	Life satisfaction				Depressive symptoms			
	*β*	SE	*β*	SE	*β*	SE	*β*	SE
Host motivation	.09	0.07	.10	0.07	−.04	0.07	−.01	0.08
Heritage motivation	.04	0.07	.07	0.07	.04	0.07	0	0.07
Host means	.12	0.08	.12	0.08	−.11	0.08	−.10	0.08
Heritage means	.13	0.07	.14	0.07	−.08	0.07	−.07	0.07
Host motivation*host means	−.12*	0.05	−.11*	0.05	.16**	0.05	.17**	0.05
Heritage motivation*heritage means	.06	0.06	.04	0.06	−.02	0.06	−.02	0.06
Host motivation*heritage means	−.10	0.07	−.11	0.07	.13	0.07	.13	0.07
Heritage motivation*host means	−.08	0.06	−.08	0.06	.07	0.06	.06	0.06
Gender (M = 1, F = 2)	—	—	−.07	0.15	—	—	−.33*	0.15
Age	—	—	0	0.07	—	—	.07	0.07
Religiosity	—	—	.05	0.07	—	—	0	0.07
Education	—	—	.14*	0.07	—	—	−.14*	0.07

Note:

* *p* < .05;

** *p* < .01.

To examine the role of belonging to the majority or minority religion in this acculturation context, we tested whether the interaction between acculturation motivations and acculturation means in predicting well‐being is qualified by the religious family background of participants—Jewish (59.8%) versus non‐Jewish (40.2%). We ran separate analyses for immigrants who identify as Jewish and immigrants who do not identify as Jewish (for regression coefficients, see Table S2 in the Supporting Information). Results revealed that for immigrants who do not identify as Jewish, the interaction between the motivation to adopt the host culture and the means for doing so predicted lower life satisfaction, *β* = −.22, *SE* = 0.10, *p* = .022, whereas no such interaction was found for immigrants who identify as Jewish, *β* = −.09, *SE* = 0.7, *p* = .18. Similarly, for immigrants who do not identify as Jewish, the interaction between the motivation to adopt the host culture and the means for doing so predicted greater depressive symptoms, *β* = .38, *SE* = 0.10, *p* < .001, whereas no such interaction was found for immigrants who identify as Jewish, *β* = .07, *SE* = 0.07, *p* = .34.

These results suggest that immigrants who have both the motivation and means for acculturating to the host culture may nevertheless experience lower life satisfaction because their religious identity precludes them from becoming fully integrated. However, these immigrants may still successfully preserve their heritage culture, and may fall back on this motivation more than immigrants who identify as Jewish. Corroborating this account, immigrants who do not identify as Jewish experienced higher life satisfaction when possessing both the motivations and means to preserve their heritage culture, *β* = .31, *SE* = 0.11, *p* = .005, relative to immigrants who do identify as Jewish, *β* = −.10, *SE* = 0.08, *p* = .17.

## 3 DISCUSSION

In Study 1, we found preliminary evidence in support of a framework of acculturation that distinguishes between motivations and means. First, we found associations between motivations in acculturation and means in acculturation. Means that facilitated the adoption of the host culture, including acquiring fluency in the host language and acquiring social contacts among members of the host culture, were associated with the motivation to adopt the host culture, but not with the motivation to adopt the heritage culture. Similarly, means that facilitated maintaining the heritage culture, including heritage language fluency and maintaining social contacts with members of the heritage culture, were associated with the motivation to preserve the heritage culture, but not with the motivation to adopt the host culture.

Second, we found that the interaction between means in acculturations and motivations in acculturation predict outcomes in acculturation, though these findings were nuanced. Specifically, the motivation for adopting the host culture predicted lower satisfaction and more depressive symptoms particularly for those with the means for adopting the host culture. Follow‐up analyses revealed that this was particularly so for a subset of immigrants—non‐Jewish immigrants who are formally and socially restricted from fully integrating into the host culture. Among this subset of immigrants, the motivation to preserve the heritage culture predicted higher life satisfaction, particularly among those with the means for doing so.

While unexpected, this result may reflect a tension common to the influence of goal pursuit on well‐being: the more motivated one is to attain a goal, the more detrimental its impact on one's well‐being when it is not attained (e.g. Mauss et al., 2011). Similarly, immigrants who have striven to integrate into Israeli society by learning Hebrew and acquiring Israeli social contacts may be most disappointed when they find that their motivation is difficult to actualize. This should particularly be the case for immigrants whose background precludes them from fully integrating into Israeli society. Specifically, while Russian immigrants were admitted to Israel for having Jewish ancestry, many originated from completely secular or inter‐married backgrounds (Tolts, 2009). The standards for admittance to Israel under the law of return were different from the standards of the Chief Rabbinate for qualifying them as Jewish. Judaism is a central part of Israeli identity, and this religious social identity can lead to the rejection of immigrants from religious minorities (Ben‐Nun Bloom et al., 2015). Even secular Israeli Jews partake in certain religious traditions, such as celebrating Bar Mitzvahs and marking holidays via family gatherings. Furthermore, the Chief Rabbinate of Israel is recognized by law as the supreme rabbinic authority in Israel and forbids marriages between Jews and non‐Jews (Triger, 2012). Consequently, Russian immigrants who do not identify as Jews or whose family background is inter‐married are both formally and socially restricted from fully integrating into Israeli society. This may be particularly damaging to the well‐being of non‐Jewish immigrants who are motivated to be Israeli and have labored to acquire the means to do so.

The nuanced findings, reflecting differences between immigrants who do or do not identify with the majority religious group, conform to the specificity principle in acculturation, in which “specific setting conditions of specific people at specific times moderate specific domains in acculturation by specific processes” (Bornstein, 2017, p. 3; see also Navas et al., 2005). Nevertheless, we contend that a motivational framework of acculturation should apply across different types of immigrants. Therefore, in Study 2, we investigated whether a motivational framework of acculturation generalizes to a sample of immigrants that markedly differ from the sample in Study 1 – immigrants to Britain from Pakistan, India, and Bangladesh.

In addition to attempting to replicate the central predictions of a motivational framework in Study 2, we also sought to replicate the moderation we found in Study 1 based on belonging to a marginalized immigrant population. The active ingredient in marginalization may be acculturative stress, to the extent that acculturative stress may result from a conflict between one's personal preferences and formal policies (Berry, 1997). Therefore, in Study 2, we explored whether acculturative stress plays a moderating role similar to belonging (or not belonging) to a marginalized immigrant population in Study 1.

## 4 STUDY 2

The purpose of Study 2 was to replicate the central findings from Study 1 in a pre‐registered study with a different immigrant population. As in Study 1, we expected motivations in acculturation to be associated only with congruent means in acculturation. In addition, we expected motivations and congruent means in acculturation to interact in predicting outcomes in acculturation. Given the moderation by religious identity in Study 1, which might be due to underlying acculturative stress, we explored acculturative stress as a potential moderator in Study 2. The pre‐registration is available at https://aspredicted.org/blind.php?x=8vd2cu.

### 4.1 Method

#### 4.1.1 Participants

The sample comprised participants who immigrated to the United Kingdom (UK) from Pakistan, India, and Bangladesh. Participants were selected from an online British panel (http://www.panelbase.co.uk/) based on indicating that they were born in one of these countries. As in Study 1, we selected participants who were no younger than 12 when they immigrated. In addition, since acculturation experiences vary by the amount of time since arrival in the host culture, we selected participants who immigrated at least five years previously.

To achieve sufficient power, our pre‐registration relied on the smallest effect size for the association between a motivation and a congruent means from Study 1 (Table 2) that was not due to a ceiling effect. A power analysis revealed that 230 participants would be sufficient to reach 90% power. This was also sufficiently powered to detect the smallest of the significant interactions between motivations and means in predicting adaptive outcomes in Study 1 (see Table S2, Supporting Information). A total of 282 participants completed the survey. Since the survey sought to assess the comprehension of the language of their heritage culture, we queried participants regarding their mother tongue at the beginning of the survey. We removed 43 participants who did not report their mother tongue, reported a bogus answer as their mother tongue, or reported English as their mother tongue. In addition, as pre‐registered, we removed three participants who completed the survey in less than one‐third of the median time, leaving 236 participants (54% female, M_age_ = 41.26, SD_age_ = 13.53).^4^

### 4.2 Materials

#### 4.2.1 Motivations and means in acculturation

Motivations and means in acculturation were assessed using the same measure from Study 1, with alterations that fit the new population: the host culture was referred to as Britain and the language of the host culture was referred to as English. The heritage culture was referred to either as Pakistan, India, or Bangladesh, depending on which country the participants indicated they emigrated from, and the language of the heritage culture was referred to as the language that participants wrote in an open response question in the beginning of the survey.^5^

#### 4.2.2 Outcomes

Outcomes in acculturation were examined using the scales assessing life satisfaction and depressive symptoms used in Study 1.

#### 4.2.3 Acculturative stress

Acculturative stress was assessed using the Riverside Acculturative Stress Inventory (RASI; Benet‐Martinez & Haritatos, 2005). The scale comprises 15 items rated on a 5‐point scale from 1 (strongly disagree) to 5 (strongly agree). Following the pre‐registration, the RASI subscale of perceived discrimination served as a covariate.

#### 4.2.4 Exploratory measures

Acculturation expectations were assessed based on the extent to which the experience of immigrating to a new culture was more difficult than participants expected it to be or easier than participants expected it to be on a 5‐point scale from 1(life in Britain has very much fallen short of my expectations) to 5 (life in Britain has very much exceeded my expectations). As in Study 1, national identity was also assessed. Additional measures were included for exploratory purposes, as documented in the pre‐registration, and are not analyzed in the results.

##### Procedure

The sample was recruited through an online survey company based in the UK. After giving consent, participants indicated in which country they were born (Britain/Pakistan/India/Bangladesh/Other), how many years ago they moved to Britain, and whether or not they were younger than 12 years old when they moved to Britain. Participants who indicated that they were born in Pakistan, India, or Bangladesh, moved to Britain at least five years ago, and did so when they were at least 12 years old could complete the survey. Next, participants reported the language of their heritage culture in an open response question. Next, participants reported their motivations in acculturation or means in acculturation in a counterbalanced order. Then, both heritage and host culture motivations, as well as heritage and host culture language proficiency, were presented in a counter‐balanced order. Next, the two outcome measures were presented in a counterbalanced order and then participants completed an attention check. Finally, participants reported their acculturative stress and demographics. Additional items were included in the survey, included national identity and expectations regarding acculturation.

### 4.3 Results

Table 4 presents the reliabilities, means, and standard deviations of the main variables and the zero‐order correlations among these variables. As in Study 1, the measure of social contacts displayed questionable reliability (*α* = .59). Removing one item to improve reliability did not alter any of the results reported below. All the heterotrait‐monotrait (HTMT) ratio of correlation values between the two motivations and four means were below .85 (Henseler et al., 2015), demonstrating discriminant validity between the study variables.

**Table 4:** Descriptive statistics and pairwise correlations among study variables (Study 2)

Variable	*α*	*M*	*SD*	1	2	3	4	5	6	7
1. Host motivation	.78	3.92	0.70							
2. Host language fluency	.92	4.53	0.64	.49**						
3. Host social network	.71	3.63	0.83	.41**	.28**					
4. Heritage motivation	.84	3.71	0.87	.31**	.22**	.02				
5. Heritage language fluency	.85	4.34	0.85	.16*	.23**	.03	.45**			
6. Heritage social network	.59	3.51	0.79	.09	.05	.11	.46**	.14*		
7. Life satisfaction	.90	4.98	1.16	.38**	.32**	.28**	.25**	.24**	.35**	
8. Depressive symptoms	.88	2.21	0.66	.12	−.10	.08	.30**	.04	.22**	−.14*

Note:

* *p* < .05;

** *p* < .01.

#### 4.3.1 Associations between motivations and means

**FIGURE 3:**
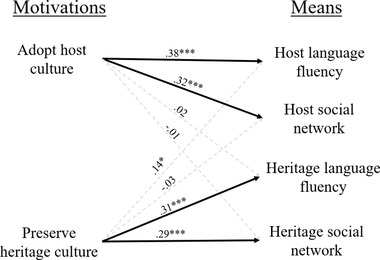
Associations between acculturation motivations and means (Study 2). Note: Values reflect standardized regression coefficients after controlling for demographic variables; *p < .05; **p < .01; ***p < .001

Zero‐order correlations revealed that the motivation to adopt the host culture is associated more strongly with the means for doing so than with the means for adopting the heritage culture. Furthermore, the motivation to preserve the heritage culture is associated more strongly with the means for doing so than with the means for adopting the host culture. To control for covariates, we regressed each motivation on the four means, the demographic variables which we controlled for in Study 1, and perceived discrimination (see Table 5).^6^ Results revealed that each motivation is associated with congruent means more than with incongruent means (see Figure 3). Motivation to preserve the heritage culture was significantly associated with the incongruent means of host language fluency (*β* = .14, 95% CI [.036, .250]), but a comparison of confidence intervals revealed that this association was significantly weaker than the two associations with the congruent means (heritage language fluency: *β* = .31, 95% CI [.210, .408]; heritage social network: *β* = .29, 95% CI [.186, .395]). In addition, a comparison of confidence intervals revealed that host language fluency was associated more strongly with the congruent motivation to adopt the host culture (*β* = .38, 95% CI [.264, .504]) than with the incongruent motivation to preserve the heritage culture.

**Table 5:** Associations between acculturation motivations and means (Study 2)

	Adopt host culture				Preserve heritage culture			
	*β*	SE	*β*	SE	*β*	SE	*β*	SE
Host language fluency	.39***	0.06	.38***	0.06	.13*	0.06	.14**	0.05
Host social network	.30***	0.06	.32***	0.06	−.07	0.05	−.03	0.05
Heritage language fluency	.05	0.06	.02	0.06	.36***	0.05	.31***	0.05
Heritage social network	.04	0.05	−.01	0.06	.41***	0.05	.29***	0.05
Gender (M = 1, F = 2)	—	—	.09	0.12	—	—	.18	0.10
Age	—	—	−.15*	0.06	—	—	−.13*	0.05
Religiosity	—	—	.05	0.06	—	—	.22***	0.05
Education	—	—	.12*	0.06	—	—	.09	0.05
Perceived discrimination	—	—	.07	0.06	—	—	.14**	0.05

Note:

* *p* < .05;

** *p* < .01;

*** *p* < .001.

#### 4.3.2 Acculturation outcomes

To test whether acculturation motivations and means interact to predict outcomes in acculturation, we regressed the two measures of well‐being on acculturation motivations, acculturation means, and their interactions. As in Study 1, we collapsed across each type of means by averaging both means associated with the motivation to adopt the host culture and both means associated with the motivation to preserve the heritage culture.^7^ We expected that positive acculturation outcomes would be predicted by the interaction of a motivation with its congruent means, but not by the interaction of a motivation with its incongruent means.

**FIGURE 4:**
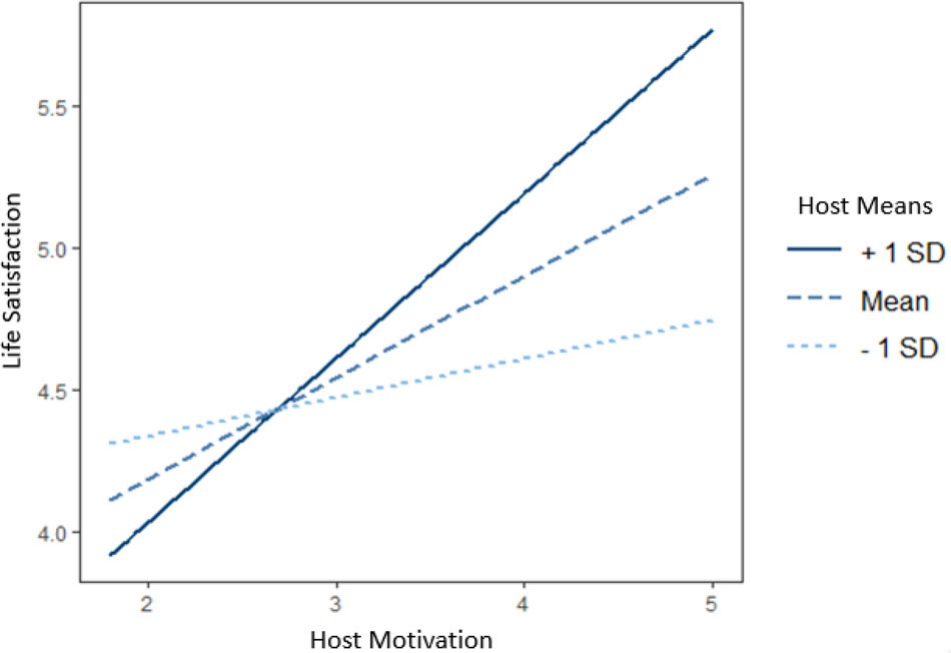
Interaction between motivation to adopt host culture and means for doing so in predicting life satisfaction (Study 2)

Results revealed one significant interaction between a motivation in acculturation and its congruent mean: the motivation to adopt the host culture and the means for doing so predicted higher life satisfaction (Table 6). An examination of this interaction reveals that the positive association between the motivation to adopt the host culture and life satisfaction was strongest for those who possessed the means for doing so (Figure 4).

**Table 6:** Predicting outcomes in acculturation via acculturation motivations and means (Study 2)

	Life satisfaction				Depressive symptoms			
	*β*	SE	*β*	SE	*β*	SE	*β*	SE
Host motivation	.25***	0.07	.21**	0.07	.04	0.08	−.01	0.07
Heritage motivation	−.05	0.08	−.08	0.08	.27**	0.08	.06	0.08
Host means	.22**	0.07	.23**	0.07	−.09	0.08	.07	0.07
Heritage means	.36***	0.07	.35***	0.07	−.01	0.08	−.01	0.07
Host motivation*host means	.12*	0.05	.13**	0.05	−.07	0.06	−.07	0.05
Heritage motivation*heritage means	.04	0.05	.03	0.05	0	0.06	0	0.05
Host motivation*heritage means	.01	0.06	.01	0.06	.12	0.07	.14*	0.06
Heritage motivation*host means	−.05	0.06	−.02	0.06	.03	0.07	−.04	0.06
Gender	—	—	−.06	0.12	—	—	.02	0.11
Age	—	—	.01	0.06	—	—	−.32***	0.06
Religiosity	—	—	.05	0.07	—	—	.05	0.06
Education	—	—	.09	0.06	—	—	−.12*	0.06
Perceived discrimination	—	—	.01	0.06	—	—	.41***	0.06

Note:

* *p* < .05;

** *p* < .01;

*** *p* < .001.

Next, we examined whether acculturative stress moderates the association between acculturation motivations and means in predicting acculturation outcomes. Acculturative stress did not moderate any associations (see Table S4, Supporting Information).

## 5 DISCUSSION

In Study 2, we replicated findings from Study 1 in support of a motivational framework of acculturation. As in Study 1, we found an association between motivations in acculturation and means in acculturation. Means that facilitated the adoption of the host culture were associated with the motivation to adopt the host culture, but not with the motivation to adopt the heritage culture. Similarly, means that facilitated the maintenance of the heritage culture were associated with the motivation to preserve the heritage culture, but not with the motivation to adopt the host culture.

Second, we found that the interaction between means in acculturation and motivations in acculturation predict outcomes in acculturation. In particular, the motivation to adopt the host culture predicted higher life satisfaction, particularly among those with the means for doing so. These findings should be interpreted with caution, however, as none of the other interactions between motivations and congruent means were significant. Overall, findings from Study 2 support a motivational framework of acculturation among immigrants from Pakistan, India, and Bangladesh to Britain, thereby corroborating the findings from Study 1 in a markedly different sample of immigrants.

## 6 GENERAL DISCUSSION

Acculturation is a motivated process, yet theoretical and empirical work in acculturation has not adequately distinguished between motivations in acculturation and means for attaining those motivations. In the present investigation, we applied theories of goal constructs (Austin & Vancouver, 1996; Kruglanski et al., 2002, 2015) to test a motivational framework of acculturation in two distinct samples. Study 1 consisted of immigrants to Israel from the former Soviet Union. Study 2 consisted of immigrants to Britain from Pakistan, India, and Bangladesh. As a type of diaspora migration, the sample in Study 1 consisted of immigrants who are less motivated to preserve their heritage culture (*M* = 3.09 on a scale from 1–5) than the sample in Study 2 (*M* = 3.71; *d* = 0.71). As a group that became a religious minority upon immigration, religiosity was a stronger motivator to preserve one's heritage culture in the sample in Study 2 (*β* = .22) than in Study 1 (*β* = −.06 for the overall sample; *β* = −.09 for Jewish immigrants and *β* = .07 for non‐Jewish immigrants). Despite the differences between samples, both studies revealed that motivations in acculturation are uniquely associated with congruent means in acculturation, and not with incongruent means in acculturation. Furthermore, both studies demonstrated the utility of accounting for both motivations and means in acculturation when predicting acculturation outcomes, even if in Study 1 this model was more relevant to a particular subgroup of immigrants and in Study 2 this emerged in a single interaction.

### 6.1 Limitations and future directions

Support for the hypothesis that specific motivations in acculturation are associated with specific means in acculturation was found across both Studies. In Study 1, the four associations between congruent motivations and their congruent means were significant, while the four associations between motivations and incongruent means were not significant (see Figure 2). In Study 2, the four associations between congruent motivations and their congruent means were significant, while three of the four associations between motivations and incongruent means were not significant (see Figure 3). The fourth association between a motivation and its incongruent mean was significant, but it was significantly smaller than the other congruent associations. Meanwhile, support for the hypothesis that motivations and means interact to predict adaptive outcomes was more equivocal. In Study 1, the interaction between motivations and congruent means predict less, not more, adaptive outcomes. Subsequent analyses established that this effect is driven by immigrants who belong to a minority religious group who are formally and socially restricted from fully integrating into the host culture. In Study 2, only one of the four interactions between motivations and congruent means were significant. Thus, further evidence is needed to evaluate whether motivations and means in acculturation do indeed interact to predict more adaptive outcomes in acculturation.

We interpreted the association between acculturation motivations and means in both studies as evidence that people pursue behaviors that facilitate the attainment of their motivations. However, an alternative interpretation is also feasible. In particular, a greater number of available means may increase motivation by increasing its salience (Kruglanski et al., 2015). According to this interpretation, it is not the motivation which leads to the pursuit of certain means, but the presence of means that enables the pursuit of certain goals. The present data cannot reconcile which account is more correct, but both accounts point to the utility of adopting a motivational framework to understand acculturation processes. Future research can tease apart the directional influence between motivations and means in acculturation via a longitudinal design assessing motivations and means both early and late in the acculturation process.

The present investigation examined two types of means in acculturation: language fluency and social contacts. However, additional means may exist, such as practicing customs and celebrating holidays, staying up‐to‐date with current events, and teaching one's children about the heritage or host cultures. Moreover, some of these means may facilitate the attainment of one acculturation motivation more than another acculturation motivation. For example, among first generation immigrants, language fluency may be more strongly associated with the motivation to adopt the host culture than with the motivation to preserve one's heritage culture, because first‐generation immigrants may have fully acquired the language of the heritage culture prior to immigrating (e.g. Birman & Trickett, 2001). Future research should examine the range of means in acculturation, and how their strength may vary by acculturation motivation.

Capturing the entire set of means for each acculturation motivation may pour light on the interplay between different means. The configuration in Figure 1, supported in Studies 1 and 2, depicts an equifinal goal systems architecture in which multiple means serve a single motivation (Kruglanski et al., 2015). Specifically, each motivation is served by two different means. In such an architecture, the means that are instrumental to a given motivation are substitutable. This means that if a given means becomes inaccessible, then pursuit of the other means will increase (Kruglanski et al., 2015). For example, consider two immigrants who are equally motivated to preserve their heritage culture. However, one immigrant lives and works among members of the heritage culture, whereas the other lives and works among members of the host culture. Given that they are equally motivated to preserve their heritage culture, the immigrant who lives and works among members of the host culture might be more likely pursue alternative means to preserve her heritage culture, such as by reading books, listening to music, and staying up‐to‐date with news from her heritage culture. Future research should examine the interplay between the pursuit of different means for attaining a given acculturation motivation.

One of the central insights of acculturation research is that acculturation consists of two orthogonal dimensions: one dimension in reference to identification, motivation, or attitudes towards one's heritage culture and another dimension in reference to identification, motivation, or attitudes towards one's host culture (e.g. LaFromboise et al., 1993; Ryder et al., 2000; Sayegh & Lasry, 1993). The distinction between motivations and means in acculturation suggests that the orthogonality of these dimensions may be nuanced. In particular, to the extent that it is possible to possess numerous motivations, motivations in acculturation may indeed be orthogonal. However, while motivations in acculturation are orthogonal, some means in acculturation may be competing. For example, when deciding where to live, immigrants may be forced to choose between living in a neighborhood with more members of one's heritage culture and living in a neighborhood with more members of one's host culture. Each of these possibilities serves different motivations, and the decision where to live must fall one way or the other. Similarly, when deciding with whom to make friends, immigrants may be forced to choose between spending time with members of the host culture versus with members of one's heritage culture. Indeed, the associations between the different means revealed different profiles: fluency for the host and heritage culture language was positively correlated in Studies 1–2, but social networks were negatively correlated (Study 1) and uncorrelated (Study 2). Consequently, it is possible that motivations in acculturation are orthogonal, but at least some means in acculturation are not. Future research should examine this question.

In Study 1, motivations and means in acculturation predicted outcomes only among a subset of immigrants from the former Soviet Union to Israel. In particular, among non‐Jewish immigrants, who are both formally and socially restricted from fully integrating into Israeli society, the motivation to integrate into the host culture interacted with the means for doing so to predict lower life satisfaction and greater depressive symptoms. Within a motivational framework of acculturation, complex relations between motivations, means, and outcomes may exist when attempts by immigrants to adopt the host culture are rebuffed. Findings from Study 2 suggests that this is not due to acculturative stress. An alternative explanation is perceived rejection by the host culture, which may lead to disidentification (Verkuyten & Yildiz, 2007). A salient context for examining this phenomenon in a different national context may be among Muslim immigrants in France, where Muslim immigrants from the Maghreb face stronger discrimination rejection than Muslim immigrants from sub‐Saharan Africa (Adida et al., 2010; Reitz et al., 2017).

### 6.2 Practical and theoretical implications

The insight that acculturation involves two independent motivational processes has been validated empirically (e.g. Ryder et al., 2000) and integrated into acculturation theory (Berry, 1997, 2003). Previous theoretical work has underlined the fundamentally motivational nature of acculturation (Gezentsvey & Ward, 2008; Toth‐Bos et al., 2019), but has not adequately distinguished between motivations and means in acculturation. The present investigation takes these insights a step further by distinguishing between two elements of motivational systems: motivations and the means for attaining such motivations. Our findings reveal that this distinction can predict the behavior of acculturating individuals, as well as their outcomes in acculturation. In particular, behaviors that facilitate the attainment of motivations in acculturation, such as immigrants’ preference for friends from the host or heritage culture, can be predicted by their motivations in acculturation. Moreover, the outcomes of immigrants in acculturation are predicted by the presence of means that facilitate the attainment of immigrants’ particular motivations. Taken together, the present investigation suggests that motivations and means in acculturation need to be more clearly distinguished in acculturation research. Such a distinction is of clear practical significance for policies and interventions addressing the incorporation of immigrants into society. Policies that seek to incorporate immigrants such as by providing them with resources must ensure that those resources match their motivations.

Many subfields in psychology investigate process that are fundamentally motivational, yet they have only recently begun distinguishing between motivations and means for attaining them. For instance, the theory of planned behavior (Ajzen, 1991) accounts for the determinants of behavior, which serve individuals’ goals, yet only recently has the theory been integrated with a motivational framework of what individuals are motivated to pursue (Ajzen & Kruglanski, 2019). The distinction between motivations and means for attaining them has provided novel theoretical and empirical insights in diverse domains, including for interpersonal processes (Orehek & Forest, 2016), emotion regulation (Millgram et al., 2019; Vishkin et al., 2020), and religion (Ben‐Nun Bloom et al., 2021; Vishkin et al., 2021). The applicability of a framework of goal constructs in explaining phenomena in disparate fields suggests that such a theoretical framework may meet the call for rigorous frameworks that cut across particular subfields and disciplines (Muthukrishna & Henrich, 2019). As new insights are gleaned regarding the properties of goal constructs in general, these insights may be further integrated into our understanding of acculturation in particular.

## Supporting information



Supporting Information

## Footnotes

1 We did not rely on structural equation modelling for this analysis because of the low number of observations relative to the parameters.

2 This association was no longer significant when using the shortened scale of Russian social contacts with higher reliability (*β* = 0.12, *t* = 1.80, *p* = .074).

3 For analyses on each of the means run separately, see Table S1, Supporting Information.

4 The pre‐registration included a rigorous attention check for monitoring data quality: requiring participants to identify 3 questions they were asked during the survey out of 7. Less than half the sample passed this rigorous quality check, and therefore it was dropped.

5 As we reported in the pre‐registration, we included novel alternative scales and stated our intention to use these scales in the event of obtaining a ceiling effect as we obtained on one of the measures from Study 1. Since no ceiling effects were obtained on the original measures, we did not analyze these novel alternative scales.

6 We decided not to control for socio‐economic status, despite pre‐registering it, because a large proportion of participants chose not to report it. Results in Study 2 remained unchanged when controlling for socio‐economic status, with exception to the comparison between the regression coefficients for heritage language fluency (*β* = .278, 95% CI [.176, .381]) and host language fluency (*β* = .169, 95% CI [.055, .283]) when predicting motivation to preserve the heritage culture, in which the 95% confidence interval of the latter overlaps with the regression coefficient of the former.

7 For analyses on each of the means run separately, see Table S3, Supporting Information.
