# Supplementary material for: A motivational framework of acculturation
Source: Brain Behav. 2021 Jun 24;11(8):e2267. doi: 10.1002/brb3.2267 (PMC8413784; doi:10.1002/brb3.2267)
Supplement: Supplementary file 1 — Supporting Information [file BRB3-11-e2267-s001.docx]

**Supplemental Materials**

Table A1. *Predicting outcomes in acculturation via acculturation motivations and specific means (Study 1)*.

*a.* *Language Fluency*

|  | Life Satisfaction | | | |  | Depressive Symptoms | | | |
| --- | --- | --- | --- | --- | --- | --- | --- | --- | --- |
|  | *β* | SE | *β* | SE |  | *β* | SE | *β* | SE |
| Host motivation | .16* | 0.07 | .17* | 0.07 |  | -.03 | 0.07 | 0 | 0.07 |
| Heritage motivation | .08 | 0.06 | .11 | 0.07 |  | .03 | 0.06 | 0 | 0.07 |
| Host means | .04 | 0.08 | .06 | 0.08 |  | -.15* | 0.08 | -.17* | 0.08 |
| Heritage means | -.03 | 0.07 | -.12 | 0.08 |  | -.08 | 0.07 | -.05 | 0.08 |
| Host motivation*host means | -.07 | 0.05 | -.05 | 0.05 |  | .12* | 0.05 | .13* | 0.05 |
| Heritage motivation*heritage means | .09 | 0.07 | .02 | 0.07 |  | -.14* | 0.07 | -.10 | 0.07 |
| Host motivation*heritage means | -.06 | 0.08 | -.08 | 0.08 |  | 0 | 0.08 | .02 | 0.08 |
| Heritage motivation*host means | -.09 | 0.06 | -.07 | 0.06 |  | .08 | 0.06 | .06 | 0.06 |
| Gender (M = 1, F = 2) | - | - | -.10 | 0.15 |  | - | - | -.32* | 0.15 |
| Age | - | - | -.03 | 0.07 |  | - | - | .05 | 0.07 |
| Religiosity | - | - | .04 | 0.07 |  | - | - | -.03 | 0.07 |
| Education | - | - | .15* | 0.07 |  | - | - | -.11 | 0.07 |

*Note.* **p* < .05; ***p* < .01.

*a.* *Social contacts*

|  | Life Satisfaction | | | |  | Depressive Symptoms | | | |
| --- | --- | --- | --- | --- | --- | --- | --- | --- | --- |
|  | *β* | SE | *β* | SE |  | *β* | SE | *β* | SE |
| Host motivation | .09 | .07 | .10 | 0.07 |  | -.10 | .07 | -.08 | 0.07 |
| Heritage motivation | .02 | .07 | .03 | 0.07 |  | .02 | .07 | 0 | 0.07 |
| Host means | .18* | .07 | .16* | 0.07 |  | -.02 | .08 | -.01 | 0.08 |
| Heritage means | .18* | .08 | .24** | 0.08 |  | 0 | .08 | -.02 | 0.08 |
| Host motivation*host means | -.12* | .06 | -.13* | 0.06 |  | .11 | .06 | .13* | 0.06 |
| Heritage motivation*heritage means | .05 | .06 | .07 | 0.06 |  | .04 | .06 | .01 | 0.06 |
| Host motivation*heritage means | -.11 | .07 | -.14* | 0.07 |  | .10 | .07 | .11 | 0.07 |
| Heritage motivation*host means | -.07 | .07 | -.06 | 0.06 |  | .06 | .07 | .06 | 0.07 |
| Gender (M = 1, F = 2) | - | - | -.08 | 0.14 |  | - | - | -.27 | 0.15 |
| Age | - | - | -.01 | 0.06 |  | - | - | .11 | 0.07 |
| Religiosity | - | - | .03 | 0.06 |  | - | - | .01 | 0.07 |
| Education | - | - | .16* | 0.06 |  | - | - | -.14* | 0.07 |

*Note.* **p* < .05; ***p* < .01.

Table A2. *Predicting outcomes in acculturation via acculturation motivations and acculturation means for Jewish and non-Jewish immigrants (Study 1).*

1. *Non-Jewish immigrants*

|  | Life Satisfaction | | | |  | Depressive Symptoms | | | |
| --- | --- | --- | --- | --- | --- | --- | --- | --- | --- |
|  | *β* | SE | *β* | SE |  | *β* | SE | *β* | SE |
| Host motivation | .05 | 0.14 | .11 | 0.14 |  | .06 | 0.14 | .07 | 0.14 |
| Heritage motivation | .04 | 0.12 | .05 | 0.12 |  | 0 | 0.12 | -.07 | 0.12 |
| Host means | -.04 | 0.14 | 0 | 0.14 |  | .12 | 0.14 | .11 | 0.14 |
| Heritage means | .26* | 0.12 | .23 | 0.12 |  | .05 | 0.12 | .09 | 0.12 |
| Host motivation*host means | -.23* | 0.10 | -.22* | 0.10 |  | .35*** | 0.10 | .38*** | 0.10 |
| Heritage motivation*heritage means | .34** | 0.11 | .31** | 0.11 |  | -.11 | 0.11 | -.14 | 0.11 |
| Host motivation*heritage means | -.14 | 0.11 | -.22* | 0.10 |  | .28** | 0.11 | .31** | 0.11 |
| Heritage motivation*host means | .05 | 0.10 | .02 | 0.09 |  | -.07 | 0.09 | -.07 | 0.10 |
| Gender (M = 1, F = 2) | - | - | -.25 | 0.24 |  | - | - | -.36 | 0.24 |
| Age | - | - | -.04 | 0.10 |  | - | - | .13 | 0.10 |
| Religiosity | - | - | -.23* | 0.10 |  | - | - | .05 | 0.10 |
| Education | - | - | .29** | 0.10 |  | - | - | -.22* | 0.10 |

1. *Jewish immigrants*

|  | Life Satisfaction | | | |  | Depressive Symptoms | | | |
| --- | --- | --- | --- | --- | --- | --- | --- | --- | --- |
|  | *β* | SE | *β* | SE |  | *β* | SE | *β* | SE |
| Host motivation | .11 | 0.09 | .08 | 0.09 |  | -.05 | 0.09 | -.01 | 0.10 |
| Heritage motivation | .05 | 0.09 | .10 | 0.09 |  | -.01 | 0.09 | -.03 | 0.09 |
| Host means | .19* | 0.09 | .20* | 0.10 |  | -.20* | 0.09 | -.19 | 0.10 |
| Heritage means | .09 | 0.09 | .11 | 0.09 |  | -.17 | 0.09 | -.17 | 0.09 |
| Host motivation*host means | -.08 | 0.07 | -.09 | 0.07 |  | .05 | 0.07 | .07 | 0.07 |
| Heritage motivation*heritage means | -.05 | 0.08 | -.10 | 0.08 |  | -.07 | 0.08 | -.07 | 0.08 |
| Host motivation*heritage means | -.16 | 0.09 | -.18 | 0.09 |  | .02 | 0.09 | .03 | 0.10 |
| Heritage motivation*host means | -.13 | 0.10 | -.13 | 0.10 |  | .18 | 0.10 | .19 | 0.10 |
| Gender (M = 1, F = 2) | - | - | .17 | 0.18 |  | - | - | -.34 | 0.19 |
| Age | - | - | .04 | 0.09 |  | - | - | .02 | 0.09 |
| Religiosity | - | - | .23** | 0.08 |  | - | - | -.03 | 0.09 |
| Education | - | - | .07 | 0.08 |  | - | - | -.10 | 0.09 |

*Note.* **p* < .05; ***p* < .01; ****p* < .001.

Table A3. *Predicting outcomes in acculturation via acculturation motivations and specific means (Study 2)*.

*a.* *Language Fluency*

|  | Life Satisfaction | | | |  | Depressive Symptoms | | | |
| --- | --- | --- | --- | --- | --- | --- | --- | --- | --- |
|  | *β* | SE | *β* | SE |  | *β* | SE | *β* | SE |
| Host motivation | .27*** | 0.07 | .26*** | 0.07 |  | .15* | 0.07 | .09 | 0.06 |
| Heritage motivation | .08 | 0.07 | .04 | 0.08 |  | .34*** | 0.07 | .08 | 0.07 |
| Host means | .12 | 0.09 | .15 | 0.09 |  | -.33*** | 0.09 | -.14 | 0.08 |
| Heritage means | .14 | 0.08 | .13 | 0.08 |  | -.14 | 0.08 | -.04 | 0.07 |
| Host motivation*host means | .04 | 0.06 | .06 | 0.06 |  | -.18** | 0.06 | -.14** | 0.05 |
| Heritage motivation*heritage means | 0 | 0.06 | -.01 | 0.06 |  | -.10 | 0.06 | -.06 | 0.05 |
| Host motivation*heritage means | .02 | 0.07 | .01 | 0.07 |  | .04 | 0.07 | .12* | 0.06 |
| Heritage motivation*host means | -.12 | 0.07 | -.08 | 0.07 |  | .03 | 0.07 | -.03 | 0.06 |
| Gender (M = 1, F = 2) | - | - | -.18 | 0.13 |  | - | - | .02 | 0.11 |
| Age | - | - | .01 | 0.07 |  | - | - | -.28*** | 0.06 |
| Religiosity | - | - | .08 | 0.07 |  | - | - | .05 | 0.06 |
| Education | - | - | .06 | 0.07 |  | - | - | -.09 | 0.06 |
| Perceived discrimination | - | - | .04 | 0.06 |  | - | - | .40*** | 0.06 |

*Note.* **p* < .05; ***p* < .01; ****p* < .001.

*a.* *Social contacts*

|  | Life Satisfaction | | | |  | Depressive Symptoms | | | |
| --- | --- | --- | --- | --- | --- | --- | --- | --- | --- |
|  | *β* | SE | *β* | SE |  | *β* | SE | *β* | SE |
| Host motivation | .34*** | 0.07 | .30*** | 0.07 |  | -.04 | 0.08 | -.04 | 0.07 |
| Heritage motivation | .04 | 0.07 | .03 | 0.08 |  | .28*** | 0.08 | .12 | 0.07 |
| Host means | .09 | 0.06 | .10 | 0.06 |  | .03 | 0.07 | .10 | 0.06 |
| Heritage means | .27*** | 0.07 | .28*** | 0.07 |  | .09 | 0.07 | .01 | 0.06 |
| Host motivation*host means | .14** | 0.05 | .14** | 0.05 |  | -.07 | 0.06 | -.09 | 0.05 |
| Heritage motivation*heritage means | .06 | 0.06 | .06 | 0.06 |  | .10 | 0.07 | .12* | 0.06 |
| Host motivation*heritage means | -.01 | 0.06 | -.01 | 0.06 |  | .06 | 0.06 | .03 | 0.05 |
| Heritage motivation*host means | .01 | 0.06 | .02 | 0.06 |  | .02 | 0.07 | .01 | 0.06 |
| Gender (M = 1, F = 2) | - | - | -.04 | 0.12 |  | - | - | .03 | 0.11 |
| Age | - | - | .04 | 0.06 |  | - | - | -.31*** | 0.06 |
| Religiosity | - | - | .04 | 0.07 |  | - | - | .04 | 0.06 |
| Education | - | - | .11 | 0.06 |  | - | - | -.12* | 0.06 |
| Perceived discrimination | - | - | -.05 | 0.06 |  | - | - | .40*** | 0.06 |

*Note.* **p* < .05; ***p* < .01; ****p* < .001.

Table A4. *Predicting outcomes in acculturation via acculturation motivations, acculturation means, and acculturative stress (Study 2).*

|  | Life Satisfaction | | | |  | Depressive Symptoms | | | |
| --- | --- | --- | --- | --- | --- | --- | --- | --- | --- |
|  | *β* | SE | *β* | SE |  | *β* | SE | *β* | SE |
| Host motivation | .24** | 0.08 | .19* | 0.08 |  | 0 | 0.07 | -.03 | 0.07 |
| Heritage motivation | -.02 | 0.08 | -.04 | 0.09 |  | .11 | 0.08 | .07 | 0.08 |
| Host means | .21** | 0.08 | .23** | 0.08 |  | .01 | 0.07 | .08 | 0.07 |
| Heritage means | .36*** | 0.08 | .35*** | 0.08 |  | .02 | 0.07 | .01 | 0.07 |
| Stress | .01 | 0.08 | .03 | 0.08 |  | .62*** | 0.07 | .55*** | 0.07 |
| Host motivation*host means | .12* | 0.05 | .12* | 0.05 |  | -.10 | 0.05 | -.09 | 0.05 |
| Heritage motivation*heritage means | .05 | 0.06 | .05 | 0.06 |  | -.02 | 0.06 | 0 | 0.05 |
| Host motivation*heritage means | -.01 | 0.07 | -.02 | 0.07 |  | .10 | 0.06 | .10 | 0.06 |
| Heritage motivation*host means | -.03 | 0.07 | 0 | 0.07 |  | -.02 | 0.06 | -.04 | 0.06 |
| Host motivation*stress | -.02 | 0.09 | -.04 | 0.09 |  | -.12 | 0.08 | -.11 | 0.08 |
| Heritage motivation*stress | .21* | 0.10 | .22* | 0.10 |  | -.05 | 0.09 | -.01 | 0.09 |
| Host means*stress | -.01 | 0.09 | 0 | 0.09 |  | .08 | 0.09 | .09 | 0.08 |
| Heritage means*stress | -.15 | 0.09 | -.15 | 0.09 |  | .01 | 0.09 | .01 | 0.08 |
| Host motivation*host means*stress | .06 | 0.08 | .04 | 0.08 |  | .03 | 0.07 | .01 | 0.07 |
| Heritage motivation*heritage means*stress | -.01 | 0.06 | 0 | 0.06 |  | -.05 | 0.05 | -.03 | 0.05 |
| Host motivation*heritage means*stress | .07 | 0.09 | .06 | 0.09 |  | .02 | 0.08 | -.01 | 0.08 |
| Host means*heritage motivation*stress | -.05 | 0.08 | -.03 | 0.08 |  | .04 | 0.07 | .06 | 0.07 |
| Gender | - | - | -.09 | 0.12 |  | - | - | .08 | 0.11 |
| Age | - | - | 0 | 0.06 |  | - | - | -.26*** | 0.06 |
| Religiosity | - | - | .06 | 0.07 |  | - | - | .03 | 0.06 |
| Education | - | - | .09 | 0.06 |  | - | - | -.11* | 0.05 |

*Note.* **p* < .05; ***p* < .01; *** *p* < .001.
